# Signaling pathways in colorectal cancer: implications for the target therapies

**DOI:** 10.1186/s43556-024-00178-y

**Published:** 2024-06-07

**Authors:** Yanlin Song, Ming Chen, Yuhao Wei, Xuelei Ma, Huashan Shi

**Affiliations:** 1 Department of Biotherapy, State Key Laboratory of Biotherapy and Cancer Center, West China Hospital, Sichuan University, No. 17, Block 3, Southern Renmin Road, Chengdu, Sichuan 610041 People’s Republic of China; 2 West China School of Medicine, West China Hospital, Sichuan University, No. 17, Block 3, Southern Renmin Road, Chengdu, Sichuan 610041 People’s Republic of China

**Keywords:** Colorectal carcinoma, Signaling pathways, Immune responses, Biomedicines, Clinical trials

## Abstract

Colorectal carcinoma (CRC) stands as a pressing global health issue, marked by the unbridled proliferation of immature cells influenced by multifaceted internal and external factors. Numerous studies have explored the intricate mechanisms of tumorigenesis in CRC, with a primary emphasis on signaling pathways, particularly those associated with growth factors and chemokines. However, the sheer diversity of molecular targets introduces complexity into the selection of targeted therapies, posing a significant challenge in achieving treatment precision. The quest for an effective CRC treatment is further complicated by the absence of pathological insights into the mutations or alterations occurring in tumor cells. This study reveals the transfer of signaling from the cell membrane to the nucleus, unveiling recent advancements in this crucial cellular process. By shedding light on this novel dimension, the research enhances our understanding of the molecular intricacies underlying CRC, providing a potential avenue for breakthroughs in targeted therapeutic strategies. In addition, the study comprehensively outlines the potential immune responses incited by the aberrant activation of signaling pathways, with a specific focus on immune cells, cytokines, and their collective impact on the dynamic landscape of drug development. This research not only contributes significantly to advancing CRC treatment and molecular medicine but also lays the groundwork for future breakthroughs and clinical trials, fostering optimism for improved outcomes and refined approaches in combating colorectal carcinoma.

## Introduction

Colorectal carcinoma stands as a prominent contributor to cancer-related mortality on a global scale, and its intricate pathogenesis involves a multifaceted interplay of various factors. Genetic mutations, dysregulated signaling pathways, and compromised immune surveillance collectively contribute to the development and progression of this malignancy [1–3]. Many signaling pathways have been involved in the pathogenesis of colorectal carcinoma, that is so complex to understand the mechanisms. For example, the EGFR-mediated signaling pathway plays a crucial role in tumor cell proliferation, that influences cellular activities, such as nuclear transcription and mitochondrial metabolism [4–6]. In addition, hyperactivation of the Wnt signaling pathway is observed in most CRC cases. Approximately 85% of these cases exhibit loss of APC function, leading to destabilization of free β-catenin [7]. CXCLs and their cognate receptors are also implicated in mediating tumor growth and metastasis [8]. Notch, typically involved in maintaining progenitor/stem cell populations, is found to be constitutively activated in colon cancers [9]. Additionally, the transcription factor STAT3 plays a significant role in forming the tumor microenvironment [10]. Transmembrane receptors, such as JMJD6 and AXL, are associated with the proliferation and motility of tumor cells [11, 12]. So many signaling pathways have confused clinicians and physicians, that commanded a brief review about this.

Various agents targeting these molecules have been developed, including traditional Chinese medicines and small molecule inhibitors, with clinical trials underway to evaluate their efficacy. The heterogeneity of CRC broadens the scope for drug development, though it complicates the selection of the appropriate therapy. Organoids, derived from human induced pluripotent stem cells (iPSCs) or human embryonic stem cells (hESCs), has been considered as a good tool for drug screening in colorectal carcinoma patients recently [13]. However, In the quest for personalized treatment, cost-effectiveness analysis is emerging as a crucial facet, optimizing therapy selection for individual patients [14, 15]. As clinical trials progress and our understanding deepens, the intricate interplay of molecular pathways in CRC becomes increasingly discernible, paving the way for refined, tailored interventions and fostering hope for improved outcomes in the battle against colorectal carcinoma.

### Current standard treatment

The pivotal role of effective first-line therapy in determining colorectal carcinoma (CRC) outcomes cannot be overstated, as clinicians navigate through a myriad of factors that shape their choices in clinical decision-making. The location of the tumor within the colorectum emerges as a crucial factor influencing therapeutic decisions [16–19]. Notably, patients with left-sided tumors stand to gain significant benefits from certain treatment modalities. This observation underscores the importance of tailoring therapies based on tumor location to optimize outcomes. Conversely, the scenario differs for patients with right-sided tumors, where the efficacy of initial epidermal growth factor receptor (EGFR)-based therapy may be limited. Beyond the initial response rate, the benefits of such therapies may not be as pronounced in this subgroup of patients. This nuanced understanding of the varying responses based on tumor location adds a layer of complexity to the selection of first-line therapies for CRC. It emphasizes the need for personalized treatment strategies that consider the specific characteristics of the tumor, ultimately aiming to maximize the effectiveness of therapeutic interventions and improve overall outcomes for individuals facing colorectal carcinoma. A worse prognosis for OS, PFS, and ORR has been observed in patients with right-sided tumor compared with those with left-sided tumor. The determination of second-line treatment hinges on the outcomes and responses observed during the administration of first-line systemic therapies, a pivotal step in the sequential management of colorectal carcinoma. Notably, anti-angiogenic agents, such as bevacizumab, emerge as frequently employed components in the second-line treatment regimen [20]. These agents, designed to inhibit angiogenesis and disrupt the tumor's blood supply, play a crucial role in extending the spectrum of therapeutic options available for patients post-first-line intervention. The utilization of anti-angiogenic agents underscores the strategic consideration of targeted therapies that address specific aspects of tumor progression, contributing to a comprehensive and personalized approach in the ongoing battle against colorectal carcinoma. The selection and customization of third-line treatments are intricately tied to the identification of specific molecular markers, thereby tailoring therapeutic approaches to the unique characteristics of the colorectal carcinoma (CRC). In cases where CRC exhibits a wild-type RAS status, the emphasis is often on utilizing kinase inhibitors designed to target and modulate specific molecular pathways associated with tumor progression [21]. This tailored strategy acknowledges the importance of aligning treatment with the molecular profile of the tumor, optimizing the chances of therapeutic success. Furthermore, in CRC cases characterized by high microsatellite instability (MSI), third-line interventions often incorporate immunotherapy as a key component. Agents such as pembrolizumab, nivolumab, or ipilimumab, known for their immune checkpoint inhibitory properties, are strategically employed to leverage the body's immune system against the tumor. This personalized approach, based on the presence of specific molecular markers, not only reflects the evolving landscape of precision medicine but also underscores the importance of leveraging targeted therapies and immunomodulation for enhanced efficacy in the management of advanced colorectal carcinoma.

The therapeutic landscape for metastatic colorectal carcinoma (mCRC) has evolved, and in 2004, a notable recommendation emerged for the management of mCRC resistant to irinotecan-based therapy. Specifically, the combination of an epidermal growth factor receptor (EGFR) antibody with irinotecan was endorsed as a strategic intervention for cases where the tumor expresses EGFR. This combination therapy represented a significant advancement, introducing a targeted approach to address resistance issues observed with irinotecan-based regimens [22]. The landscape of metastatic colorectal carcinoma (mCRC) witnessed a significant development in 2006 with the approval of panitumumab, a fully human IgG2 monoclonal antibody, for third-line treatment in cases where the tumor expresses epidermal growth factor receptor (EGFR) [23]. This milestone marked a substantial stride in the quest for effective and targeted therapies tailored to the molecular characteristics of mCRC. Panitumumab might offer a more effective long-term treatment option for mCRC patients compared to cetuximab [24, 25]. Unfortunately, intrinsic and extrinsic resistance limits its use in colorectal cancer. Various strategies, including new EGFR-targeted inhibitors, combination therapies, novel cytotoxic drugs, and metabolic regulators, have been employed to overcome this resistance [26]. Bevacizumab, a humanized anti-VEGF monoclonal antibody, demonstrated promising preclinical and clinical efficacy against CRC, especially when combined with chemotherapy, leading to its approval in 2004 [27]. To counter the resistance of bevacizumab, targeting tumor cell-derived CCL2, a candidate gene involved in ETV5 + colorectal cancer, has been identified. The combination of bevacizumab with an anti-CCL2 antibody has shown a synergistic effect in inhibiting tumor growth and angiogenesis [28].

The effectiveness of EGFR (epidermal growth factor receptor) and VEGFR (vascular endothelial growth factor receptor) inhibitors faces limitations in KRAS wild-type colorectal carcinoma (CRC). In cases where the KRAS gene is wild-type, the use of inhibitors targeting EGFR and VEGFR pathways has demonstrated a certain level of efficacy. However, it is essential to acknowledge that neither cetuximab (an EGFR inhibitor) nor bevacizumab (a VEGFR inhibitor) has exhibited superior efficacy in the context of first-line treatment among patients with KRAS-mutated metastatic colorectal carcinoma (mCRC) [29], while combinations such as EGFR inhibitors with PARP, which is a family of proteins involved in a number of cellular process, such as DNA repair and genomic stability, inhibitors (olaparib) have effectively curbed tumor growth in KRAS-mutant cancer cell-derived xenograft models [30]. Vigilant consideration of adverse effects is paramount in the comprehensive evaluation of treatment options for colorectal carcinoma. Notably, the combination of XELIRI (capecitabine plus irinotecan) with bevacizumab, a regimen that has demonstrated promising response rates, demands careful attention due to the prevalence of certain side effects, most notably neutropenia, in patients undergoing this treatment [31]. Clinical evidence has demonstrated that pegfilgrastim plays a pivotal role in diminishing the incidence of grade 3/4 febrile neutropenia (FN). Pegfilgrastim, a form of granulocyte colony-stimulating factor (G-CSF), stands out as a proactive therapeutic intervention designed to address the heightened risk of FN, particularly in individuals undergoing treatments associated with a high risk of neutropenia. [32].

### EGFR mediated signaling transduction

Aberrant activation of the epidermal growth factor receptor (EGFR), characterized by dysregulated cascade reactions, plays a pivotal role in the uncontrolled formation of tumor masses and the release of cytokines. This aberration in EGFR signaling pathways provides a crucial window of opportunity for disease control, making EGFR antibodies a cornerstone in the standard therapy for colorectal carcinoma (CRC) patients. However, the persistent challenge of resistance has restricted the widespread application of EGFR antibodies over the years. In response to resistance mechanisms, a paradigm shift in therapeutic strategies has been witnessed. The introduction of ATP-competitive and covalent binding inhibitors is aimed at targeting downstream molecules within the intricate signaling network, including RAS-RAF-MEK-ERK and PI3K-AKT-mTOR pathways. By addressing these downstream components, therapeutic interventions seek to overcome resistance and re-establish control over the tumor's growth and survival mechanisms. Understanding the intricate biological effects of these pathways assumes considerable significance in the quest to enhance the efficacy of clinical treatment for CRC. Insights into the crosstalk and interplay within these signaling cascades provide a foundation for developing targeted therapies that can navigate around resistance mechanisms. This elucidation becomes a cornerstone in refining treatment strategies, offering the potential for more effective interventions and improved outcomes in the dynamic landscape of colorectal carcinoma management.

#### RAS-RAF-MEK-ERK implications in colon cancer

The downstream signaling pathway, RAS-RAF-MEK-ERK, constitutes a crucial component of the extensive serine-threonine kinase family, exerting profound influence on various aspects of cellular behavior. This intricate cascade plays a multifaceted role in key cellular processes, including cell proliferation, metastasis, angiogenesis, and extracellular matrix degradation [33]. EGFR, also known as ERBB, a glycoprotein with intracellular tyrosine kinase and extracellular protein ligands, but the ERBB receptors are subfamily of four tyrosine kinase receptors including EGFR, HER2/neu, HER3, and HER4 (https://www.ncbi.nlm.nih.gov/books/NBK482459/). Upon the binding of epidermal growth factor (EGF) to the epidermal growth factor receptor (EGFR), a cascade of molecular events is initiated at the membrane's inner face. This intricate process involves the recruitment of growth-factor-receptor bound protein 2 (GRB2) and son of sevenless (SOS) to the membrane, marking a critical step in the activation of the GTP-binding protein RAS. This molecular signaling pathway plays a pivotal role in transducing extracellular signals into the cell, ultimately influencing key cellular processes. In recent years, there has been a substantial expansion in our understanding of the intricacies of this signaling pathway, particularly in the context of colorectal carcinoma (CRC). The molecular events triggered by EGFR activation and the subsequent activation of RAS have been scrutinized with increased precision. This heightened comprehension extends beyond the basic mechanics of the pathway and delves into the specific alterations and dysregulations that characterize colorectal cancer [34]. Aberrant activations within cellular signaling pathways have been identified as key contributors to pathological conditions, and this includes dysregulation in the epidermal growth factor receptor (EGFR), RAS, and BRAF. The intricate interplay of these components often leads to abnormal signaling cascades within the cell. In particular, the downstream effector, extracellular signal-regulated kinase (ERK), has been implicated in aberrant activation, representing a significant facet of these perturbed signaling pathways [35]. The influence of aberrant activations, including EGFR, RAS, and BRAF, extends beyond the initial signaling events to impact downstream molecules crucial for cellular regulation. Notably, downstream effectors such as cyclin D1, MMP1, P53, and c-MYC are intricately influenced by these dysregulated signaling pathways [36].

Mutations in the RAS gene family, encompassing KRAS, HRAS, and NRAS, represent a prevalent genetic alteration in colorectal carcinoma (CRC). Among these, KRAS mutations are particularly noteworthy, being identified in over 30% of CRC patients. Within KRAS mutations, there is a notable association with malignancy development, especially when occurring in codon 12 [37]. Another noteworthy mutation in colorectal carcinoma (CRC) involves the BRAF gene, which serves as a downstream molecule in the RAS signaling pathway. The mutation in BRAF represents a significant alteration and is among the most prevalent mutations observed in CRC [38]. While RAF is recruited by activated RAS, the activation mechanism of BRAF differs significantly, primarily taking place through a distinctive process involving dimerization with various forms of RAF. Unlike the straightforward activation process seen in other RAF family members, BRAF activation is intricately linked to the formation of dimers, representing a unique regulatory mechanism within the RAS-RAF-MEK-ERK signaling pathway [39]. MEK and ERK, downstream of RAF, are activated by RAF through phosphorylation, leading to the activation of ERK, mitogen-activated protein kinase (MAPK). Activated ERK regulates gene expression in the nucleus with the aid of transcription factors, including c-FOS, c-JUN, S6, and others. The signaling transduction is more complex than described, involving intricate pathways like MAPK, which includes ERK1/2, JNK, and P38. The function of c-FOS can be regulated by both JNK and ERK, occasionally by H-RAS [40].

**Fig. 1:**
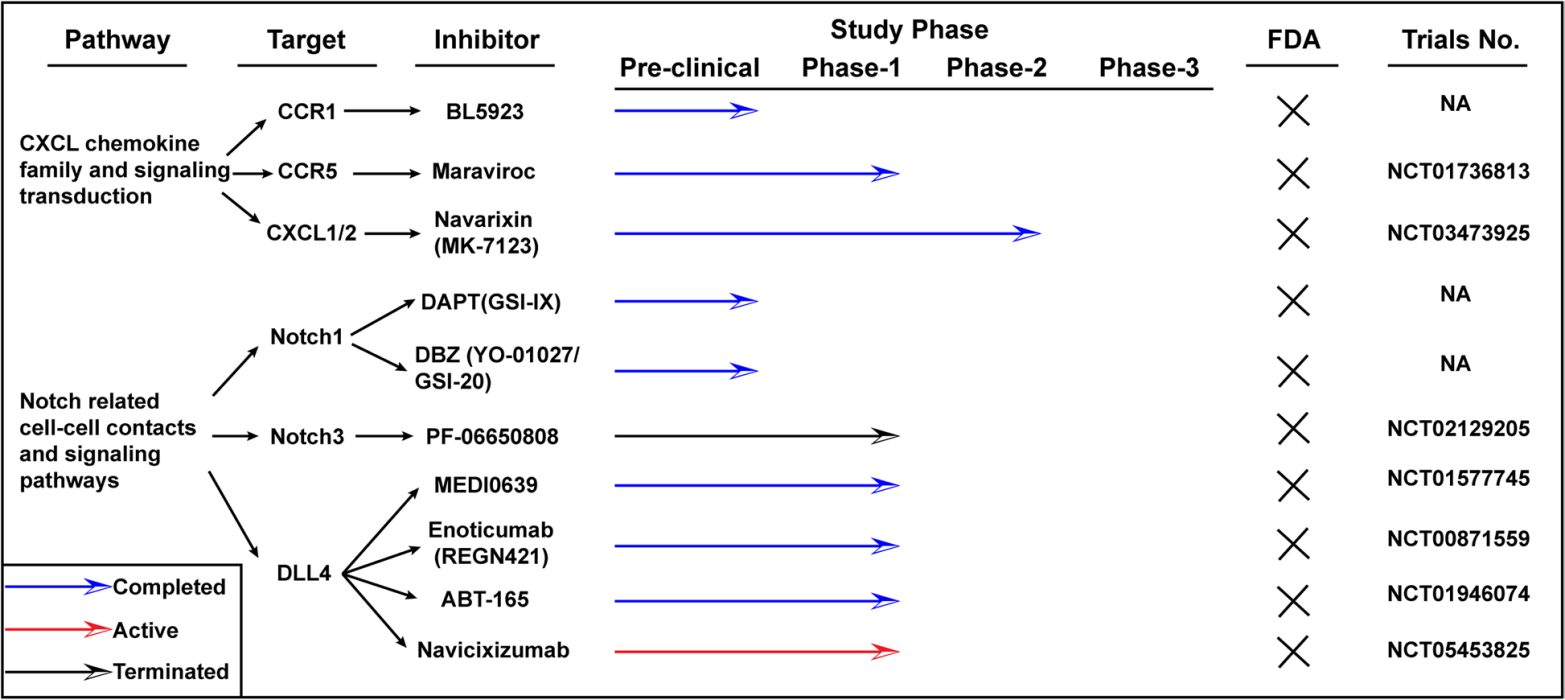
Clinical information of inhibitors targeting CXCL/Notch involved signaling transduction. The information is limited in colon cancer

Despite the development of EGFR antibodies, such as cetuximab and panitumumab, as targeted therapies for colorectal carcinoma (CRC), the clinical outcomes have presented challenges. The disease has shown progression in over 50% of patients treated with these EGFR antibodies, indicating the complexity of overcoming resistance mechanisms in CRC. KRAS mutations, found in 40–50% of patients, unsurprisingly diminished cetuximab's efficacy [41, 42]. RAS, a GTP-binding protein, has long presented a formidable challenge in therapeutic targeting due to its elusive nature. Despite extensive efforts, identifying effective targets for RAS inhibition has proven to be a complex task. However, recent advancements have seen the development of several inhibitors specifically designed to target a distinct mutation within the KRAS gene known as KRAS^G12C^. The synergistic potential of combining anti-BRAF and anti-EGFR therapies has demonstrated noteworthy effectiveness in the management of metastatic colorectal carcinoma (mCRC) patients harboring the BRAF^V600E^ mutation, particularly those who have undergone prior treatment with an anti-EGFR inhibitor [43]. Encorafenib combined with cetuximab is reported as the new standard care for BRAF^V600E^ mCRC [44, 45]. Adding an MEK inhibitor to this regimen does not extend survival but increases the objective response rate (ORR) and toxicity. A recent study reveals that a chimeric receptor, CD16-158-valine on T cells, overcomes cetuximab resistance in KRAS mutant CRC, which indicates a new direction of CRC treatment, the combination of immune and molecule targets [46]. Other clinical trials have been summarized (Fig. 1), that phase 3 clinical trials have been activated in RAS inhibitors while those has been terminated in MEK and RAF inhibitors. Clinical trials in ERK inhibitors have limited in phase 1 clinical trials, which verified the safety but command much attentions.

#### PI3K-AKT-mTOR is involved in tumor cell growth, proliferation, and differentiation

PI3K/Akt/mTOR, are crucial kinases that control essential functions of cells. Various biological molecules, such as epidermal growth factors, sonic hedgehog, IGF-1, insulin, and calmodulin, activate this signaling pathway. Other molecules antagonize this pathway, including phosphatase and tensin homolog (PTEN), glycogen synthase kinase 3β, and transcription factor HB9 [47]. The PI3K/Akt/mTOR pathway exerts a profound influence on a myriad of biological processes, playing a pivotal role in the regulation of cell survival, growth, proliferation, and tumor angiogenesis. This intricate signaling cascade is integral to the maintenance of cellular homeostasis and is frequently implicated in the development and progression of various malignancies, including colorectal carcinoma [48–50]. The PI3K/Akt/mTOR pathway plays a crucial role in the initiation and progression of colorectal cancers, where factors like circIL4R and metabolites enhance CRC cell proliferation, migration, and invasion through the PI3K/Akt signaling pathway [51–53]. Selectively inhibiting PI3K/Akt isoforms increases the sensitivity of human carcinoma cell lines to radiotherapy, chemotherapy, and growth factor deprivation stress [54–59].

**Fig. 2:**
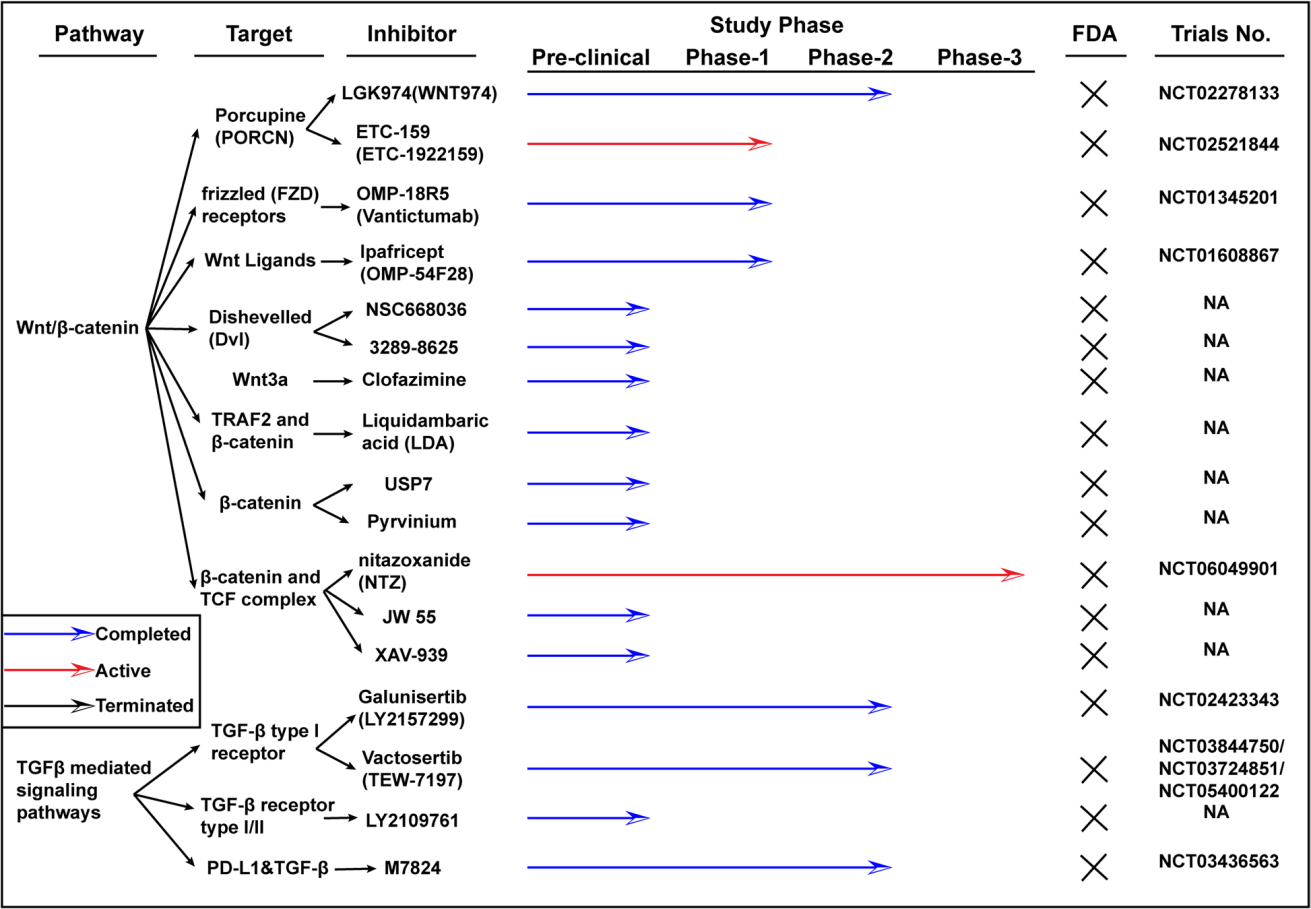
Clinical information of inhibitors targeting Wnt/β-catenin and TGFβ involved signaling transduction. The information is limited in colon cancer

Duvelisib and umbralisib (Fig. 2) are globally approved PI3K inhibitors used for treating relapsed or refractory lymphoma [60, 61], while Alpelisib is used for treating PI3K-mutated, HR ( +) advanced breast cancer [62]. Idelalisib and copanlisib, FDA-approved drugs, are used for various cancers [63, 64]. However, adverse effects reported include acute liver injury, anorexia, diarrhea, nausea, hyperglycemia, mucositis, pruritus, and rash, among others [65–67], and about 30% of patients have skin problems. MTOR inhibition plays a role in particulate matter-induced bronchial epithelial inflammation, with autophagy being a key factor in this process [68]. Extensive exploration of the precise mechanism is necessary for making personalized therapeutic decisions. In addition, the recommended phase II dose (RP2D) of PI3Kinase inhibitor in combination with panitumumab was established in patients with KRAS wild-type CRC [69]. The combination of MAPK and PI3K/Akt inhibitors is suggested as MAPK signal inhibition alleviates autophagy-related psoriasis [70]. Autophagy contributes to intestinal homeostasis, shielding the host from harmful inflammation and dysfunctional mitochondria through estrogen-related receptor α-influenced gut microbiota [71].

Natural products targeting the PI3K/Akt pathway have recently attracted significant interest. Salidroside, derived from Rhodiola Rosea, induces autophagy and apoptosis in colorectal cancer cells. Celastrus orbiculatus demonstrates a similar effect [72]. Scutellariae Radix and Coptidis Rhizoma regulate glucose and lipid metabolism through the MAPK/PI3K/Akt signaling pathway [73]. Bufalin, a compound used in traditional Chinese medicine and extracted from “Chan Su,” serves as an anti-tumor agent. Long-term, low-dose bufalin administration effectively suppresses tumorigenesis by inhibiting the PI3K/Akt pathways and C-X-C motif chemokine ligands like CXCL1, CXCL2, and CXCL5 [74].

#### Combinations of EGFR antibodies

Combining EGFR antibodies with small molecular inhibitors offers advantages such as enhanced inhibition efficacy, resistance overcoming, and improved outcomes. Dual targeting of EGFR's extracellular and intracellular domains may enhance treatment efficacy and overcome resistance typical of single-agent therapies [75, 76]. For instance, EGFR TKIs remain effective even after patients develop resistance to EGFR antibodies, and vice versa. EGFR antibodies, like cetuximab and panitumumab, target the receptor's extracellular domain, preventing ligand binding and receptor activation [77, 78], while small molecular inhibitors, like erlotinib and gefitinib, act intracellularly, inhibiting EGFR kinase activity [79, 80]. However, brivanib alaninate (a VEGFR inhibitor) did not significantly improve OS with cetuximab in wt-KRAS CRC patients. In KRAS wild-type cases, various strategies have been developed to overcome cetuximab resistance, targeting CD137 [81], CD51 [82], and insulin-like growth factor type-1 receptor (IGFR-1) [83], and capsule of these factors [84], single-walled carbon nanotubes [85], liposomes. In contrast, MEK inhibitors, either alone or combined with EGFR inhibitors, show promising outcomes. MEK1/2 inhibitors (AS703026 and AZD6244) are potential approaches for KRAS-mutated colorectal cancer resistant to EGFR monoclonal therapy [86, 87]. Validating these findings is further needed for the benefit of colorectal cancer patients with KRAS mutations in research and clinical trials [88, 89]. In addition, other combinations are also explored, like toll-like receptor agonists [90, 91], MEK and PI3K/mTOR inhibitors [92], BRAF inhibitors [93], and VEGF(R)-targeted small molecules [94] to overcome resistance and improve therapeutic responses*.*

### Signaling pathways involved in the fate of colonic epithelial cells

#### Molecular pathological changes based on the colonic epithelial cells

The colonic architecture comprises four layers: the mucosa, submucosa, muscle layer, and serosa, with the mucosa itself subdivided into epithelium, lamina propria, and a thin muscular layer. Typically, the epithelium contains gel-like mucins and columnar cells, characterized by a high turnover rate, crucial for eliminating invasive particles. Mucins, large and highly glycosylated proteins, play a vital role in protecting the luminal surface of the gastrointestinal tract [95]. These mucins coat the apical surface of enterocytes and are secreted by goblet cells. The colonic epithelial layer, a complex and dynamic structure, comprises various cell types such as goblet cells, enterocytes, enteroendocrine cells, and intestinal stem cells. Notably, it lacks Paneth cells, specialized anti-microbial molecule-producing cells, found in the small intestine [96]. Every cell type within the colonic epithelium is pivotal in regulating various aspects of colonic physiology, such as mucosal formation, colonic absorption, colonic motility, immunity, and regeneration, and these cells are all derived from colonic stem cells. However, this normal function is disrupted when bacteria compromise the barrier integrity, leading to epithelial aberrations with genetic mutations. This disruption stimulates immune cells to release cytokines, promoting tumor cell growth [97].

**Fig. 3:**
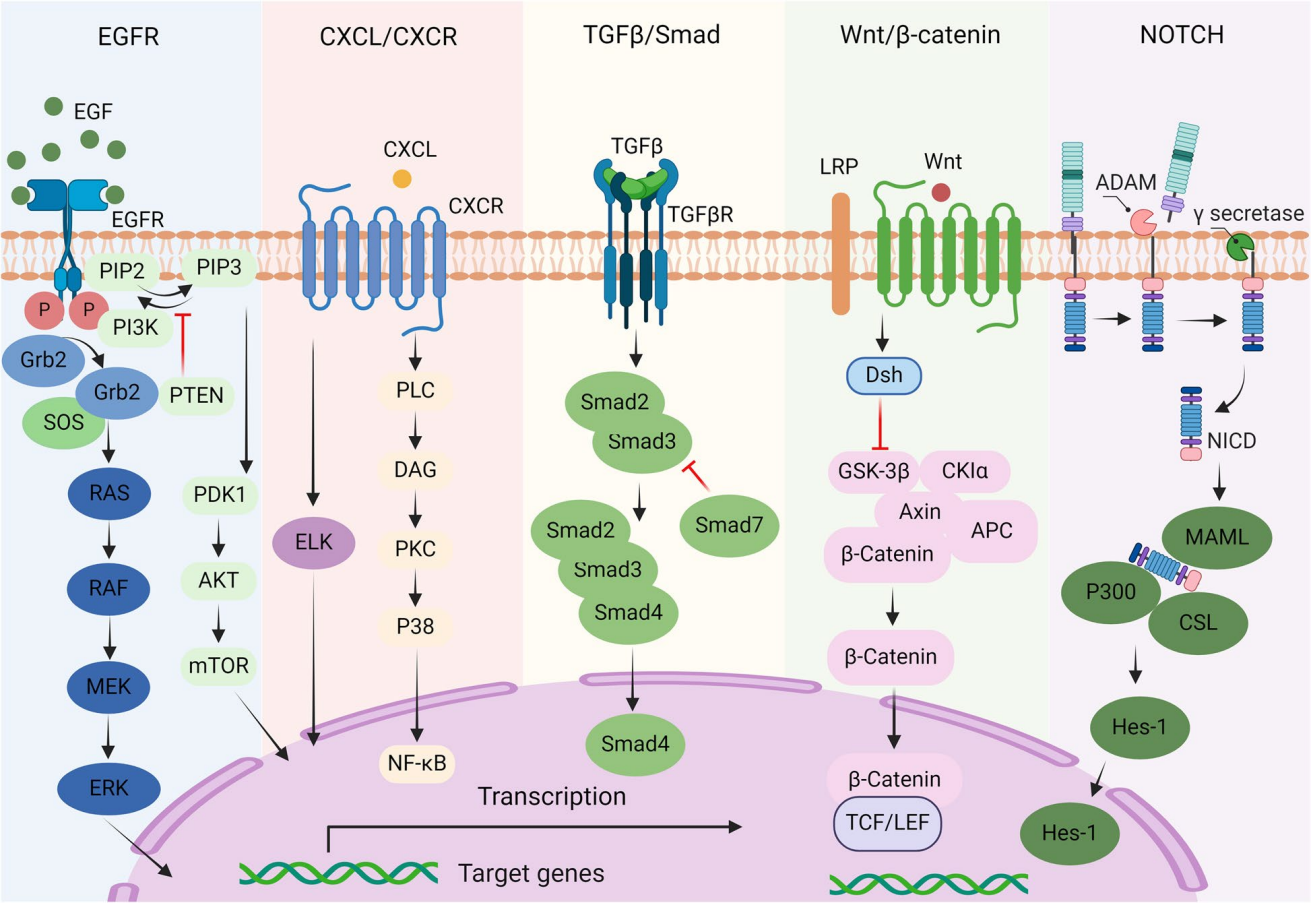
Signaling pathways involved in colorectal cancer. These pathways play important roles in cell growth, proliferation, and differentiation, thus, mutations of them may influence normal functions of cells, leading to stem-like genotypes. These pathways include (from left to right) the Notch, EGFR/MAPK, PI3K/Akt, CXCL, TGFβ, and Wnt pathways. ADAM, a disintegrin and metalloproteinase; NICH, cleaved form of Notch; CSL, CBF1, suppressor of hairless, Lag-1; MAML, mastermind-like proteins; HES-1, hairy and enhancer of split 1, DNA binding protein; EGF, Epidermal growth factor; EGFR, Epidermal growth factor receptor; PI3K, phosphatidylinositol 3-kinase; PTEN, phosphatase and tensin homologue; PIP2, phosphatidylinositol 4,5-bisphosphate; PIP3, phosphatidylinositol (3,4,5)-trisphosphate; PDK1, 3-phosphoinositide dependent protein kinase 1; AKT, protein kinase B; mTOR, mammalian target of rapamycin; SOS, son of sevenless; GRb2, growth factor receptor-bound protein 2; RAS, one type of small G-proteins; RAF, down-stream of RAS, MAPKKK; MEK, down-stream of RAF, MAPKK; PLC, phospholipase C; PKC, protein kinase C; DAG, diacylglycerol; SMAD, drosophila mothers against decapentaplegic protein; Dsh, phosphoprotein dishevelled; GSK3, glycogen synthase kinase-3; CK1, casein kinase 1

The particular bacteria species consist mainly of two types, Bacteroides fragilis and Escherichia coli, among 500 different types of bacteria [98]. The subtype of B. fragile called enterotoxigenic Bacteroides fragilis (ETBF) releases a toxin, Bacteroides fragilis toxin, that triggers certain oncogenic [99]. The E. coli strain produces a substance called colibactin that causes DNA mutations. Intriguingly, mice with colons colonized by only one of these species develop few or no tumors. In contrast, simultaneous colonization with both species results in numerous tumors, indicating a synergistic interaction between the two bacteria types. The inflammatory protein IL17 is crucial in ETBF-induced tumor formation. Deleting the IL17 gene prevents tumor development in mice [100]. Additionally, the ETBF toxin triggers a series of events promoting colon inflammation, which in turn affects the colon epithelial cells through pathways like Wnt/β-catenin, TGFβ/STAT3, and CXCL/NF-κB (Fig. 3) [101, 102].

#### Cytokines and transcriptional factors which are involved in the tumorigenesis

NF-κB and STAT3 are key factors that link inflammation to cancer, likely due to their close association with the tumor-associated inflammatory environment. NF-κB triggers the expression of numerous pro-inflammatory genes, encoding cytokines and chemokines, like TNF and interleukins (ILs), and is also involved in inflammasome regulation [103]. Additionally, NF-κB plays a crucial role in regulating the survival, activation, and differentiation of innate immune cells and inflammatory T cells. Chronic activation of STAT3 leads to increased expression of cell cycle and apoptosis-related proteins (Cyclin D1, c-MYC, and survivin) and decreased expression of anti-apoptotic proteins like Bcl2 and Mcl1 [104]. Currently, STAT3 inhibitors are developed to target the SH2 domain, DNA-binding domain, and N-terminal domain [105, 106]. Napabucasin, recognized as a leading cancer stemness inhibitor, has shown good tolerance in Japanese patients [107, 108].

IL17, a proinflammatory cytokine, also identified as CTLA8, released by macrophages and Th17 cells, contributes to the invasion and metastasis of CRC cells via NF-κB signaling [109]. IL17 stimulates the production of IL6, an inflammatory cytokine, and IL8, a chemokine ligand for CXCR2, which mediates neutrophil recruitment to tissues [110]. Moreover, IL6 from tumor-associated macrophages (TAMs) activates the JAK/STAT3 pathway, leading to transcriptional inhibition of tumor suppressors [111], while caspase-related IL-8 is considerably related to the recruitment of anti-inflammation macrophage through neutrophil [112]. TNFα plays a role in colon cancer angiogenesis through the TNFα/NF-κB signaling pathway [113]. Overexpression of TGFβ and IL10R2 enhances IL22/STAT3 signaling in colitis-associated colorectal carcinogenesis [114, 115], and Wnt5a-mediated IL10 secretion from macrophages ultimately promotes tumor growth and metastasis [116]. Neutralizing IL10 significantly increases the proportion of CD8 + T cells, leading to enhanced tumor cell death [117].

#### CXCL chemokine family and signaling transduction in tumor and immune cells

Chemokine (C-X-C motif) ligands, CXCL1-17, comprise a vast family, with some members serving as prognostic biomarkers in colorectal carcinoma [118]. These chemokines are pivotal in modulating inflammation, cell proliferation, angiogenesis, and immune responses via chemokine receptors CXCR1 to 8. CXCL4, the first discovered member of the chemokine family by Walz and colleagues, is also known as platelet factor 4 [119]. CXCL8, or IL8, produced by macrophages, epithelial cells, and endothelial cells, transmits signals through PI3K and MAPK pathways via CXCR1/2 [120]. Besides, CXCL8 is associated with dendritic cell activation through some markers, including CD80, CD86, and CD83 [121]. Knockdown of SMAD4 in human colorectal carcinoma cells leads to increased expression of CXCL1 and CXCL8, indicating cross-talk between TGF and CXCL signaling pathways and neutrophil recruitment [122]. CXCL13 correlates with elevated T follicular helper cells and innate cells in the tumor microenvironment, binding to CXCR5 on the cell membrane [123]. Likewise, CXCL13 and CXCL1 foster tumor development via the AKT signaling pathway [124, 125], additional chemokines such as CXCL14, CXCL3, and CXCL12 serve as predictive factors [126–128]. In CRC metastasis, two distinct CXCL12 pathways are identified: CXCL12-CXCR4 and CXCL12-CXCR7 axes, with CXCL directly influencing metastasis through the Wnt-β-catenin signaling pathway [129].

**Fig. 4:**
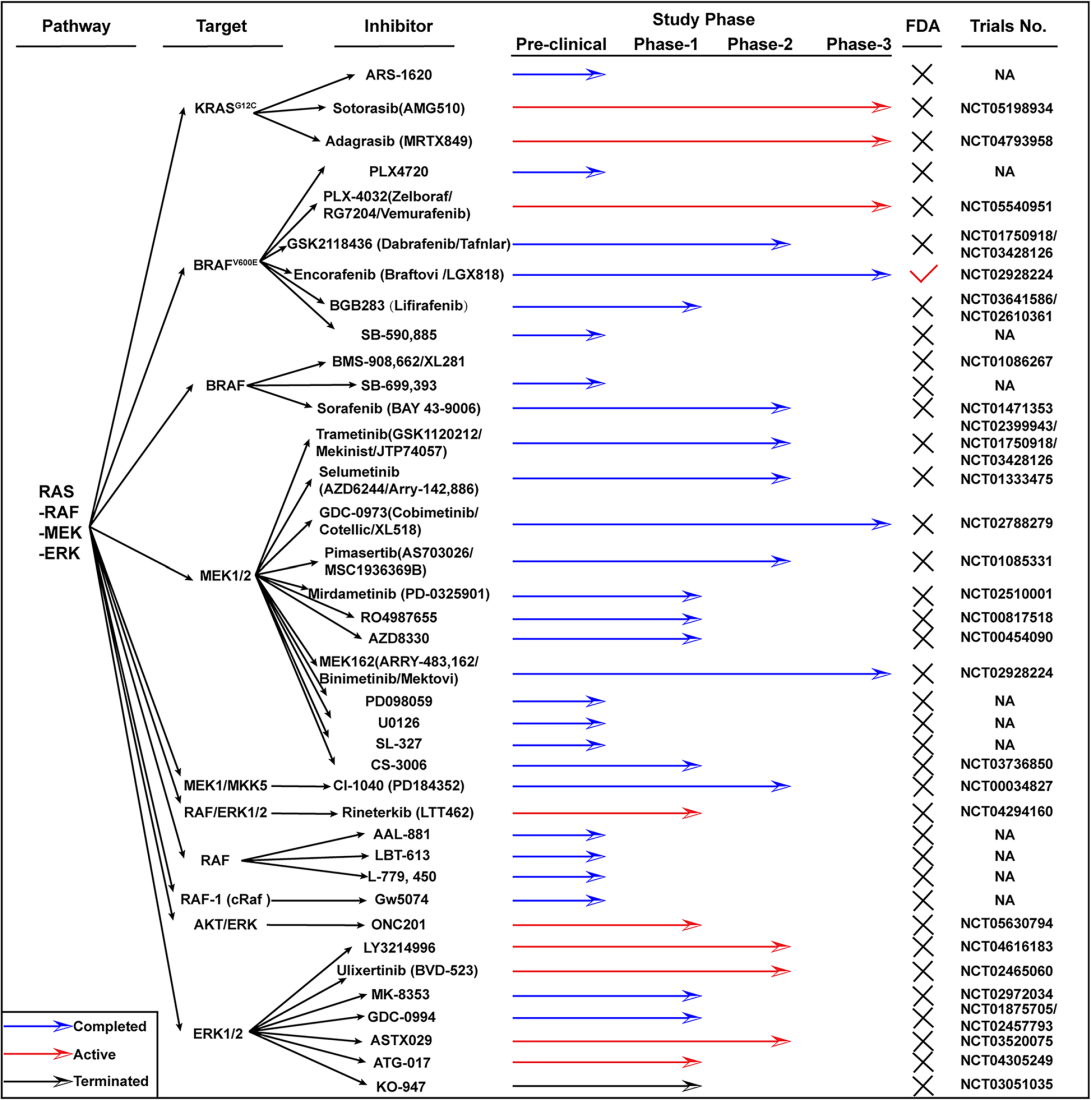
Clinical information of inhibitors targeting MAPK signaling pathway. The information is limited in colon cancer

Targeting CXCR, specifically the frizzled transmembrane Ca2 + signaling pathway, demonstrates significant potential in CRC treatment, encompassing CCR1, CCR5, and CXCR2 (Fig. 4). CCR1 antagonists curb CRC progression by targeting CCR1-expressing myeloid cells [130]. CCR5 shows high expression in both primary and liver metastatic masses, associated with a reduced cytotoxic/regulatory T cell ratio and diminished M2 macrophage polarization [131]. Maraviroc, a CCR5 inhibitor, curtails T cell recruitment to colorectal carcinoma and hinders liver metastasis in advanced refractory CRC [132, 133]. The safety and efficacy of pembrolizumab and maraviroc have been explored in phase 1 clinical trial (NCT03274804) [134]. A phase 2 clinical trial is investigating the combination effects of Navarixin, a CXCL1/2 inhibitor, with pembrolizumab in advanced microsatellite CRC (NCT03473925) [135]. Although many targeted inhibitors are used for treating hematological diseases, no CCR or CXCL inhibitors have been have been approved by FDA yet.

#### Notch-related cell–cell contacts and signaling pathways

The Notch signaling pathway, a key cellular communication system, regulates cell proliferation, differentiation, and apoptosis [136–138]. This pathway comprises Notch receptors (Notch1-4), ligands (Jagged1-2, DLL1, DLL3, DLL4), and numerous downstream genes such as p21 and hes-1 [139]. In healthy colonic tissues, Notch signaling plays an essential role in maintaining intestinal epithelial integrity [140]. Immunohistochemistry studies reveal elevated Notch1 and Jagged1 expression in colorectal carcinoma and adenoma compared to adjacent carcinoma and normal tissues [141]. Notch signaling influences intestinal stem cell fate, steering them towards differentiation into secretory cells, potentially explaining the increased expression in adenocarcinomas [142]. Within colorectal cancer, Notch signaling fosters tumor growth and impedes differentiation by downregulating targets like math1 [9]. RT-PCR analysis indicates high Notch1 expression in colorectal carcinoma cell lines [143]. Silencing Notch1 with siRNA arrests cells in the G1 phase and promotes apoptosis [144]. Notch1 activation is associated with a doubling in metastasis rates, predominantly liver metastases, due to TGFβ-dependent neutrophil recruitment in KrasG12D-driven serrated cancers [145].

The primary method of inhibiting the Notch pathway currently involves gamma-secretase inhibitors (GSIs, Fig. 4), which fall into two categories: those binding to the active catalytic site of presenilins and those targeting a different site [146]. Preclinical studies indicate that inhibiting Notch signaling can improve the survival of cells treated with chemotherapeutic agents such as 5-fluorouracil (5-FU) and oxaliplatin [147]. A key therapeutic target in the Notch pathway is the Notch receptor, where γ-secretase inhibitors obstruct the production of the oncogenic intracellular domain of Notch molecules, thereby inhibiting Notch activity [148]. Initially, gamma-secretase inhibitors were developed for Alzheimer's disease research, targeting the production of beta-amyloid peptides from amyloid precursor protein (APP) [149]. Clinical development of these inhibitors was halted due to adverse effects related to Notch cleavage inhibition in the brain and skin. Several companies are investigating GSIs for treating Notch-dependent malignancies, potentially reducing the need for extended treatments [150]. GSIs disrupt the final proteolytic step in Notch activation, blocking the release of the Notch intracellular domain (NICD) [151]. Thus, these drugs are theoretically pan-Notch inhibitors. As gamma-secretase cleaves numerous substrates (over 90), GSIs lack specificity to Notch alone [152].

The Notch pathway plays a role in the epithelial to mesenchymal transition (EMT) process, also influencing endothelial migration [153, 154]. Inhibiting DAB1/Dab1, a gene activated by Notch signaling, reduces tumor invasion in colon cancer models [155]. Elevated Notch expression levels negatively predict overall survival (OS), with Notch1 positively correlating with tumor stage in histological findings [156, 157]. Crosstalk between Notch and Wnt pathways has been observed; Notch inhibition promotes liver-specific tumor growth by upregulating the Wnt/β-catenin signaling pathway and enhancing pro-tumor cytokine production from Kupffer cells, similar to tumor-associated macrophages [158]. Notably, diverse roles of Notch have been noted in immune cells. Notch2 deficiency reduces the anti-tumor efficacy of dendritic cells (DCs), impacting T cell-mediated anti-tumor responses such as differentiation, migration, and antigen cross-presentation to CD8 + T cells [159]. DAPT, an orally active GSI, enhances CD8 + T cell cytotoxicity and pro-inflammatory cytokine secretion while reducing PD-1 expression in colorectal carcinoma patients [160].

Similar effects to those observed with DAPT have been noted in mutations of the Notch signaling pathway [161]. Notch4 mutations show favorable responses to immune checkpoint inhibitors [162]. DAPT decreases Notch1 and Hes-1 expression, enhancing radiosensitivity in CRC [163]. DBZ (YO-01027/GSI-20) increases cell death in platinum-treated CRC cells [164]. CB-103, a pan-Notch inhibitor, selectively targets the Notch intracellular domain (CSL-NICD) with a manageable safety profile and biological activity in adenoid cystic carcinoma patients [165]. Compounds like LY3039478, PF-03084014, and AL101, targeting pan-Notch, are under investigation for Notch-dependent cancers. Other GSIs, including BMS 299897 and LY-411575, are currently limited in bench research. IMR-1, an inhibitor targeting the Notch transcription complex, prevents Maml1 recruitment to the chromatin-bound Notch ternary complex (NTC) and inhibits transcription of Notch target genes [166]. IMR-1A, the acidic metabolite of IMR-1, exhibits increased potency compared to IMR-1. Other inhibitors of this signaling pathway, undergoing clinical evaluation, include PF-06650808 (NOTCH3, phase 1), MEDI0639 (DLL4, phase 1), Enoticumab (DLL4, phase 1), ABT-165 (DLL4, phase 1), and Navicixizumab (DLL4, phase 1).

#### Wnt/β-catenin, a conserved signaling pathway determining the fate of cells

The Wnt/β-catenin signaling pathway is a canonical signaling pathway without selective therapeutic agents fifteen years ago [167]. Wnt ligands are lipid-modified glycoproteins [168], and work by binding with seven-pass transmembrane protein Frizzled receptors [169], and regulate crucial cellular processes, including motility, polarity, and proliferation during embryonic development and stem cell renewal [170–172]. Constitutive activation of the pathway is found in almost all CRC patients [173–175], such as key component mutations, like APC, β-catenin, and ect [176, 177]. LRP5/6 act as co-receptors of Wnt ligands and are indispensable for Wnt signal transduction [178]. This pathway is also composed of the β-catenin destruction complex (AXIN, APC, GSK-3β, and CK1), and the transcriptional machinery involving TCF/LEF [179]. In the absence of Wnt, β-catenin is maintained at low levels through a proteolytic turnover [180]. Upon activation, the signaling transfer to APC/Axin/GSK-3β/CK1α/β-catenin complex through Dsh, and Axin works as a scaffold protein and directly binds each of the components in this complex. Then the accumulated β-catenin enters the nucleus and acts as a co-factor of transcription [181]. Notably, the GSK3β is inhibited by Dsh in this process.

Cancer stem cells (CSCs), a small subset of undifferentiated tumorigenic cells capable of self-renewal and generating diverse cell clones, are implicated in resistance to standard therapies and tumor relapse [182]. The loss of APC, a negative regulator of the Wnt/β-catenin pathway, leads to its constitutive activation, maintaining CSC survival and promoting clonal exclusion of WT-ISC through the secretion of Wnt antagonists in the tumor microenvironment (TME) [183]. The Wnt signaling pathway is multifaced that stem-like CD8 + T cells/TCF + CD8 + T cells are necessary for long-term maintenance of T cell responses and immunotherapy [184, 185], while aberrant activation of Wnt signaling pathway hinders T cell-mediated anti-tumor immune response [186]. Stem-like CD8 + T cells initiated from lymph nodes were co-stimulated from antigen-presenting cells in the tumor microenvironment. And abnormal activation of Wnt signaling pathways alters a number of regulators critical for the antitumor activities of T cells [187]. Generally, CD8 + T cells kill tumor cells through activation and maintain of memory T cells. TCF1, transcriptional factor of Wnt signaling pathway, also named as T cell factor, expresses highly in undifferentiated CD8 + T cells and memory CD8 + T cells, and depletion of TCF1 promotes effector CD8 + T cells differentiation and expansion [188]. Meanwhile, Wnt signaling pathway is significantly related with spontaneous activation of T cells and apoptosis of mature naïve CD8 + T cells partially due to c-MYC, Wnt target gene [189]. Insufficient assistant of Th cells could lead to failure of the anti-tumor immune response, that involving cytokines, co-stimulation molecules and generation of memory CD8 + T cells. Wnt signaling pathway also regulates the development and function of regulatory T cells [190].

**Fig. 5:**
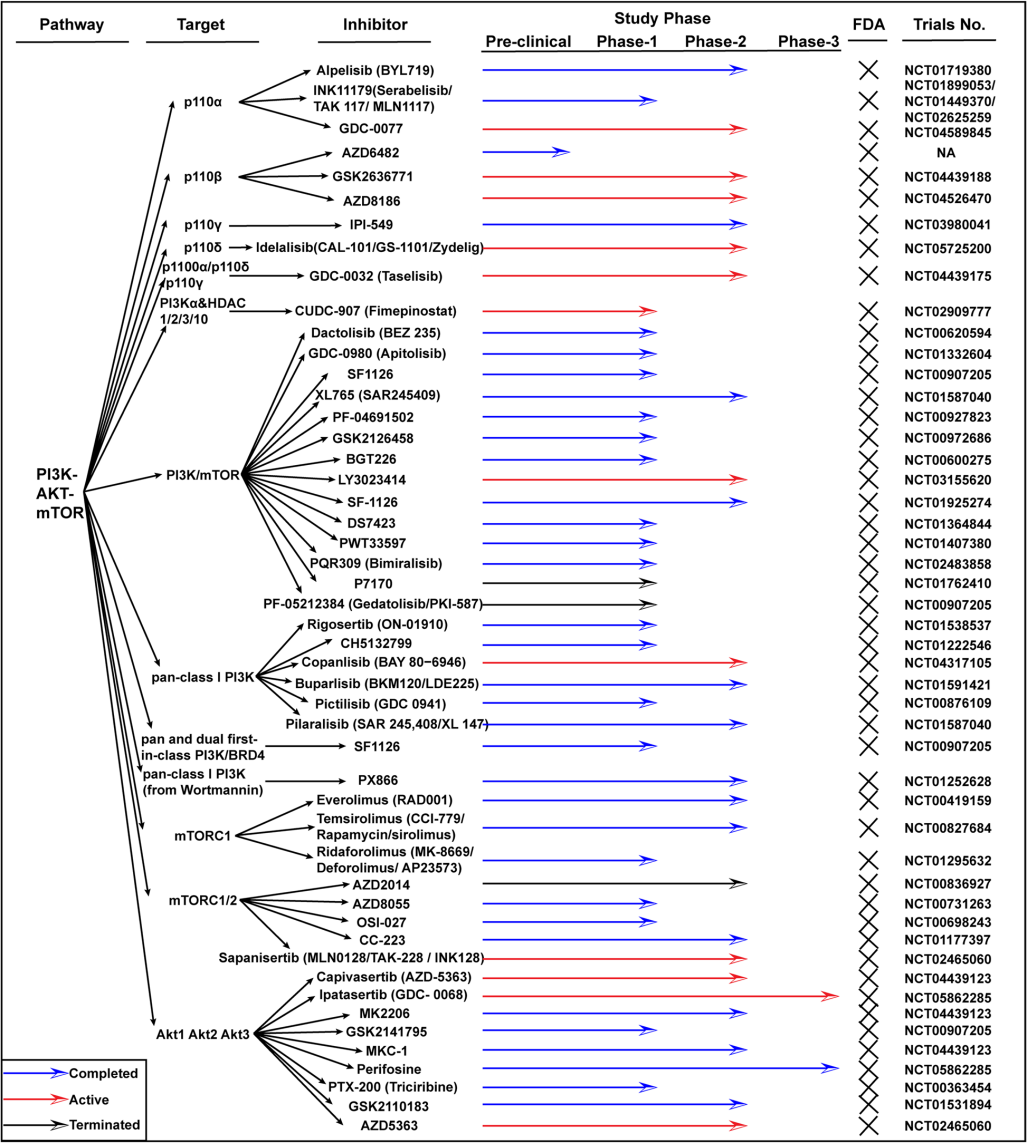
Clinical information of inhibitors targeting PI3K-AKT-mTOR signaling pathway. The information is limited in colon cancer

Drugs of the Wnt signaling pathway include natural compounds (Vitamin D, lycopene, and Genistein), existing drugs (sulindac, celecoxib, and NSAIDs), newly developed targeted therapies, and natural products (resveratrol and silibinin) [191]. Inhibitors have been developed to target mild Porc, Tnks (poly-ADP polymerase), CK1, Gsk3, Dvl, Tnik, β-catenin-Lef/Tcf complex, and transcription co-factor (Fig. 5). Porcupine (Porc) inhibitors, such as LGK974 and ETC-159, have shown efficacy in preclinical models and are currently undergoing clinical trials [192], and they target PORCN, a membrane-bound O-acyltransferase crucial for Wnt ligand secretion. Monoclonal antibodies against specific Wnt ligands or receptors, like OMP-18R5 (Vantictumab) [193] and the first-in-class recombinant fusion protein Ipafricept (OMP-54F28), have been evaluated in phase 1 clinical trials [194]. OMP-18R5 is being tested for safety and efficacy in combination with chemotherapy in several cancer types. Ipafricept has shown promising results in patient-derived ovarian cancer xenografts, decreasing the frequency of stem cells and enhancing the efficacy of chemotherapy [195]. ETC-159 has a synergistic effect with nivolumab in a preclinical trial [196]. The combination of pembrolizumab and ETC-159 is well tolerated in a phase 1B clinical trial (NCT02521844). LGK974 blocks Wnt3a production and is evaluated in phase 1 clinical trial (NCT01351103) [197]. DVL inhibitors are also Wnt secretion inhibitors, such as NSC668036 and 3289–8625, that the frontier one is an inhibitor targeting Wnt3a, which prevents TGF-induced fibroblast proliferation, migration, and differentiation [198], while another one promotes multiple drugs’ toxicities by silencing the DVL [199]. Clofazimine inhibited the production of Wnt3a by binding the GTP after the Wnt-FZD interaction, which was verified in CRC cell lines [200]. Liquidambaric acid (LDA), a natural compound, disrupts the interaction between TNF receptor-associated factor 2 (TRAF2) and the 'undruggable' N-terminal region of β-catenin, a novel approach to inhibit the Wnt/β-catenin signaling in CRC [201]. Other inhibitors work by β-catenin degeneration (AXIN stabilizers or CK1 agonists) and targeting β-catenin and TCF complex, such as NTZ [202], JW55 [203], and XAV-939 [204].

Immunotherapies, including immune checkpoint inhibitors, CAR-T, cancer vaccines, are effective treatment options for tumor patients, but the promising outcomes only occur in a limited number of cancers [186]. Wnt signaling pathway regulated behavior of tumor cells and immune cells in cancer immunotherapies. Studies have elucidated the meaning of combinations that inhibitors of Wnt/β-catenin combined with ICIs significantly inhibited the growth of renal adenocarcinoma in mouse model. Also, a clinical trial evaluating the combination of the STAT3/β-catenin signaling inhibitor, BBI608 with pembrolizumab, is ongoing in CRC patients (EPOC1503) [205]. In addition, Wnt/β-catenin releases the resistance of T-cell exhaustion which is one challenge of CAR-T. Many antigens for colorectal cancer have been identified, such as carcinoembryonic antigen (CEA), MUC1, and guanosine cyclooxygenase C (GUCY2C, GCC) [206], but they fail to demonstrate sufficient clinical efficacy because of immune escape phenomena which indicates for the combination with inhibitors of Wnt/β-catenin. Combinations that including the vaccines and inhibitors have been verified in mouse and clinical trials, that indicating a direction of CRC research.

#### TGFβ mediated signaling pathways

Transforming growth factor beta (TGF-β), initially discovered in the culture medium of virus-transformed mouse fibroblasts, plays a complex role in CRC [207], including three isoforms in mammals (TGF-β1, -β2, -β3) [207]. The mediated signaling pathway is involved in cellular processes, including growth, survival, migration, fate specification and differentiation. Recently, the signaling is initiated by the binding of TGF ligands and receptors, type I and II are transmembrane serine/threonine kinases [208], while type III receptor regulates the binding of TGF-β to type II receptor [209]. SMAD proteins, transcriptional factors, which consist of two conserved domains, MH1 and MH2, are found to bind DNA sequence and transcriptional coactivators respectively [210]. TGF-β induces G1 arrest which is committed to executing DNA replication [211, 212], while multiple strategies induce cell cycle arrest through blocking the TGF-β mediated signaling pathway [213, 214]. In CRC, TGF-β's function is dualistic and stage-dependent [215]. Initially, it suppresses tumor growth by inducing apoptosis in early-stage epithelial cells, especially those with oncogenic RAS mutations. However, in advanced stages, cancer cells often evade TGF-β's suppressive effects, leading to tumor progression, invasion, and metastasis. The pathway is a master regulator of epithelial-to-mesenchymal transition, crucial for metastasis [216]. Its overactivation and the signaling pathways mediated by its receptors play a crucial role in the onset and development of malignant tumors, making a focus on small molecule inhibitors targeting TGF-β and its receptors increasingly. In addition, TGF-β regulates B cell development and function for autoimmunity, that hematopoiesis stem cells are under high pressure of TGF-β in fetal liver and bone marrow [217]. Inhibiting of TGF-β suppresses the fibrosis in CRC [218, 219].

TGF-β/SMAD signaling pathway is one of the most prevalent alterations involved in the drug resistance of CRC [220]. Many factors regulate the resistance of chemotherapies through the TGF-β/SMAD signaling pathway [221]. TGF-β1 is more abundantly expressed in the tumor microenvironment (TME) compared to its other subtypes, contributing to resistance against immunotherapy [222]. Although no drugs targeting TGF-β have been approved, combinations have been evaluated preclinic and in clinic (Fig. 5). TGF-β inhibitor (LY2157299) and AXL inhibitor significantly reduce migration capabilities of human CRC cells [223]. Vactosertib (TEW-7197), a small molecule inhibitor specifically targeting the TGF-β type I receptor, is under clinical trial for dose escalation study [224]. TGF-β kinase inhibitor, LY2109761, inhibits liver metastases of CRC in a TGF-β inducible reporter system [225]. Clinical trials evaluating the efficacy and safety for the combination with chemotherapy, radiotherapy. and immunotherapy are active (NCT02688712, NCT02947165, NCT03436563) [226, 227]. Vactosertib, potentially improved the anti-tumor efficacy of 5-Fluorouracil in colon cancer [228], and the its combination with ICIs is under the clinical evaluation (NCT03724851) [229, 230]. A clinical trial combining Interleukin-2 (IL2) with Vactosertib is currently recruiting patients (NCT05400122) [231].

### Cost-effectiveness analysis

While clinical trials have assessed treatment efficacy, the identification of the most effective systemic treatment for colorectal cancer remains uncertain. Specifically, questions regarding the safety and benefits of sequential treatments remain unresolved. A Markov model, designed for cost-effectiveness analysis, has been employed to assess the risks and benefits of existing sequential systemic treatments in randomized clinical trials (RCTs) [232, 233]. The incremental safety effectiveness ratio (ISER), a novel metric, has been introduced to determine the optimal sequence of cetuximab-based combinations, integrating key outcomes like life-years gained and severe adverse events (SAEs ≥ 3 grade). Prospective cost-effectiveness analysis indicates that cetuximab, combined with standard chemotherapy, is an effective treatment for metastatic colorectal cancer, dependent on factors such as patient subgroups and individual responses [234]. Concurrently, mutation testing, which informs treatment decisions, has also been identified as a cost-effective factor [235]. This testing aids in assessing the economic viability of different interventions and guides decision-makers in allocating healthcare resources [236, 237]. Despite its significant cost, detecting KRAS and BRAF status is beneficial due to the clinical value of these tests [238].

The positive response of some patients to treatment, even in the absence of EGFR mutations, underscores the need for further research into treatment mechanisms [239]. Bevacizumab, however, is likely to be more cost-effective than panitumumab and cetuximab in KRAS-WT mCRC [240]. Capecitabine combined with bevacizumab is not likely a cost-effective option for elderly mCRC patients [241, 242], though similar benefit/risk profiles are observed across different age groups [243]. Collectively, these findings underline the need for a holistic approach to colorectal cancer treatment, encompassing precision medicine through mutation testing, thorough cost-effectiveness analysis, addressing treatment challenges, strict compliance with drug labeling, adaptation to emerging treatment strategies, and acknowledgment of the complexity in treatment responses and adverse events [244, 245].

### Colonic organoid, a model of the large intestine in cancer targets drug-validating

**Fig. 6:**
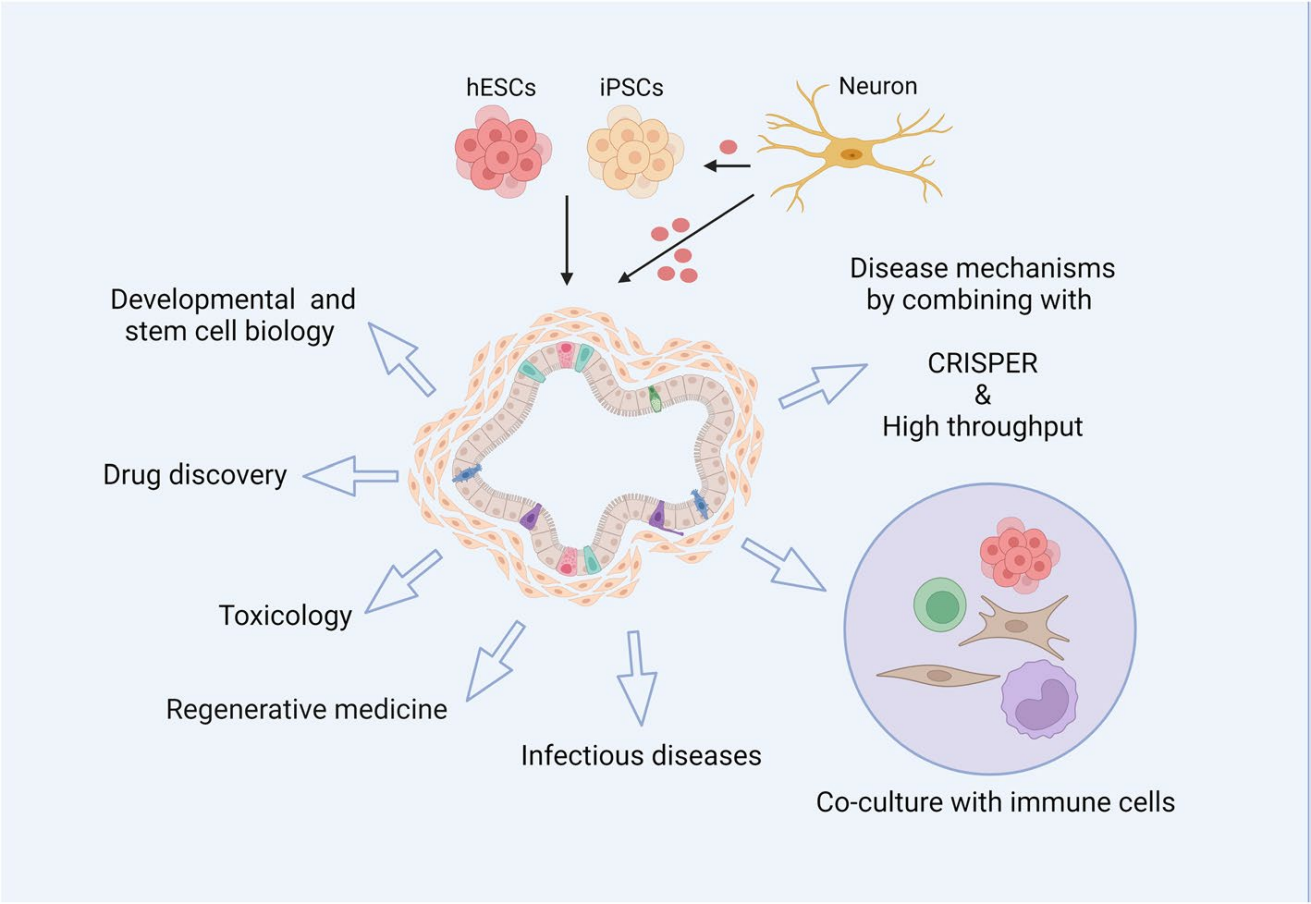
Application of colonic organoid. Colonic organoid is initially applied for developmental and stem cell biology study. In colon cancer, it is used for drug discovery and toxicology, and disease mechanisms exploring which is generally combining with high throughput and CRISPER. The colonic organoid is also co-cultured with immune cells. Besides, the organoid is also used in regenerative medicine and infectious diseases

Organoid development was initially from developmental biology, which was focused on self-patterning events and morphogenetic rearrangements for decades, but it was used for disease modeling and drug tests recently (Fig. 6) [246]. Two cell lines, human embryonic stem cells (hESCs) and induced pluripotent stem cells (iPSCs) have been used to generate colonic organoid [247]. Peripheral neuron-organoid interactions play roles in the development of colonic epithelial cells, specifically of colon enterochromaffin cells, an inter-organ interaction in organ development [248], and non-synaptic substance P which not only contains in cerebrospinal fluid but also the cells of peripheral tissue, plays an important role during this process [249, 250].

Colonic organoid reveals epigenetic vulnerabilities and is a good model for drug validation at the level of individual patient [251, 252], also, organoids provide an affordable tool for the validation and prediction of targeted therapies [253]. CRC organoid has been established as a biobank to explore the efficacy of single and combined use of different inhibitors in RAS/RNF43-mutant CRC [254–256], the repurposing drugs [257], and identify the biomarkers predicting sensitivity to EGFR inhibitors [258]. The combinations of Hedgehog signal inhibitors (AY9944 and GANT61) with traditional chemotherapies decreased the cell viability, as well as inhibited tumor cell stemness, decreased the organoid colony formation, and reversed the drug resistance, compared with Notch and Wnt signal inhibitors [259]. The co-inhibition of IL11/STAT3 signaling by drugs increases the MHC-1 expression and T cell infiltration in intestine organoid [260]. The combination of organoid and high throughput has been used to guide precision medicine [261] and reveal the mechanism of diseases, such as colitis and cancer [262].

Meanwhile, organoids may predict chemotherapy response that a phase III clinical trials implied that similar responses to chemotherapies have been observed between patients and patients derived organoids [263], including 5-fluorouracil and oxaliplatin [264]. A colonic organoid is applied to investigate the relapse that a gene, Mex3a, which contributes to the metastatic outgrowth, is identified, accompanied by Wnt downregulation and transient reminiscent of YAP, while Mex3a-deficient cells are unable to resist after chemotherapy [265]. The combination of organoid and CRISPER technology has been used to identify factors in CRC, such as the drivers of resistance to TGFβ mediated tumor restriction and suppressors in human CRC [266, 267], while colonic organoid has been used for evaluating the cytotoxicity of CAR [268].

In addition, a coculture system of tumor organoid-T cells has been established for the evaluating of T-cell-based immunotherapy in non-small lung cancer [269, 270], an artificial thymic organoid system has been used to explore the development of T cells [271], and patient-derived organoid has been used to analyze personalized T cell response to neoantigens [272]. Similarly, a coculture system with monocytes, CD8 + T cells, and patients derived tumor organoids was used to study the relationship between CD8 + T cells and tumor cells, but contrary trend between CD8 + T cells and tumor associated macrophages has been observed that is involved in CXCR4/CXCL12 pathway [273], and colonic organoid is used to explore the resistance to immune check blockage, which is accompanied by a peritoneal metastases [274], and organoid is also used to epigenetically analyzed the MHC-II which is repressed in CRC [275]. Another combination, the co-culture with cancer-associated fibroblasts, is also used to study the feature of colon cancer [276].

## Future perspectives

Colorectal carcinoma (CRC) stands out as an advanced disease characterized by high morbidity, and the identification of effective therapeutic targets remains a pivotal challenge in its treatment. The intricacies of CRC present hurdles, and the current landscape is marked by limitations in the efficacy of molecular-targeted therapies, largely attributed to issues of resistance and off-target effects. Recognizing the critical need for advancements in CRC treatment, this study delves into potential signaling pathways based on the collective research efforts of numerous investigators. By reviewing a spectrum of studies, the research outlines several targets and pathways that hold promise for consideration in the quest for more effective therapeutic strategies. Among these, the EGFR/VEGFR, CXCL/CXCR, Wnt/β-catenin, and Notch pathways are highlighted as potential avenues for targeted intervention. The study systematically captures the progress of drugs targeting these pathways, presenting a comprehensive overview through summarized tables. This synthesis of current research endeavors and the compilation of potential targets underscore the dynamic nature of ongoing efforts to unravel the complexities of CRC biology and improve treatment modalities. As the understanding of molecular pathways advances, this study provides a valuable resource for researchers and clinicians navigating the evolving landscape of CRC therapeutics, with the ultimate goal of enhancing patient outcomes in the face of this challenging and heterogeneous disease.

FDA-approved EGFR/VEGFR targeted agents have been used over 15 years. While these agents have shown good efficacy, resistance often develops, potentially due to reactivation of the pathway or bypass mechanisms [277]. Many strategies have been designed to conquer these obstacles, such as tyrosine kinase inhibitors (reversible and irreversible), gefitinib, and erlotinib [278–282]. Notably, anti-VEGF monoclonal antibodies, improving overall survival in fluoropyrimidine-based combinations but not in oxaliplatin-based therapies for stage II, have been approved for CRC treatment. Sequential bevacizumab administration showed no improvement in ORR and fewer adverse effects, but offered an overall survival advantage and improved health-related QOL [283, 284]. In addition, bevacizumab-based neoadjuvant therapy enhanced outcomes in mCRC patients without elevating complication rates, and its addition to oxaliplatin-based chemotherapy lessened splenic enlargement and thrombocytopenia occurrences, which indicated the potential of antiangiogenic treatments to traditional chemotherapies [285, 286]. Bevacizumab-based therapies are presently employed as second-line treatments for mCRC. From an economic perspective, bevacizumab is less costly compared to panitumumab + fluoropyrimidine-based chemotherapy (FBC) or cetuximab + FBC [240].

Targeting the RAS-RAF-MEK-ERK/PI3K-AKT-mTOR pathway is viewed as a strategy to overcome resistance to EGFR and VEGFR antibodies. Many inhibitors have been developed and achieved good efficacy preclinic and in clinic. The combination of EGFR antibodies with small molecular inhibitors offers a compelling strategy enhancing the effectiveness of EGFR-targeted therapies in CRC. Further research and clinical trials need to optimize treatment regimens and identify ideal patient populations, the potential for synergistic effects and resistance mitigation underscores the significance of this therapeutic approach in the evolving landscape of cancer treatment.

Wnt/β-catenin is considerable for therapeutic intervention but limited by the toxic effects associated with on-target activity on normal cells [191]. Several nuclear β-catenin effectors raise hope for their effective therapeutic window [173], but the temporal, spatial, or compositional features are still challenges. Increasing studies highlight the role of natural products in managing tumor cell metabolic disorders via the Wnt/β-catenin signaling pathway. Although Notch and Wnt strongly interact, their roles vary based on the microenvironment. Mutations in Notch contribute to anti-tumor immunity through the regulation of CD8 + T cells, macrophages, and dendritic cells, while inhibition of Notch by the DAPT inhibitor enhances the cytotoxicity of CD8 + T cells. Notch is involved in the terminal differentiation of CD8 + effector T cells, priming of naive CD8 + T cells, and activation of effector T cells [287–289]. A specific Notch inhibitor markedly reduces PD-1 expression and inhibits Pdcd1(programmed cell death 1) transcription, without impacting T-cell division, explaining why anti-tumor effects do not occur [290]. Another study verifies that conditional transgenic expression of Notch-1 leads to a central memory phenotype and enhances cytotoxicity (high granzyme B levels) [291]. The ongoing controversy regarding Notch's role in CD8 + T cells could be attributed to their potential reprogramming into memory stem cells with significant anti-tumor effects [292]. The complex role of TGF-βunderscores the potential for targeted therapies in CRC. Inhibitors of the TGF-β signaling pathway, particularly in combination with immune checkpoint inhibitors, are the focus of current research. Understanding the interplay between TGF-β and the tumor microenvironment, including cancer-associated fibroblasts and immune cells, may lead to novel therapeutic strategies.

Organoids offer a three-dimensional model that closely mimics the architecture and function of real tissues, allowing for the observation of pathophysiological changes in a controlled environment, while conventional methodologies fall short in emulating the nuanced physiological conditions and dynamic interplays intrinsic to living systems. These cancer organoids generated from 3D have emulated the structural and functional attributes of native tissues, facilitating an unprecedented examination of pathophysiological transformations within a meticulously controlled setting. Colonic organoids derived from iPSCs which model colorectal cancer are used for drug testing, and MEK inhibition has been associated with significant morphological changes in colorectal cancer organoids [293]*.* The inhibition leads to a cystic reorganization of the organoid structure, which is accompanied by an increase in LGR5 expression, an indicative marker of stemness in colorectal cancer. This suggests that MEK inhibition may induce a shift towards a stem cell-like phenotype within the organoids. The differentiation of colonic epithelial cells which underscores the interplay between neural influences and epithelial dynamics [294]. However, differences were observed in tumor and organoid, the intestinal organoid from RNF43 knockout mice did not show continuous growth, but the tumors were markedly larger in knockout mice than in wide-type mice [295]. In addition, colonic organoid is only part of the adenoma-carcinoma sequence, that difference is observed between colonic and adenoma tissues [296].

## Conclusions

This comprehensive study provides a detailed overview of the historical advancements in EGFR/VEGFR antibodies and delves into recent progress concerning several signaling pathways intricately involved in the tumorigenesis of colorectal carcinoma (CRC). While FDA-approved drugs targeting these pathways have been integral to clinical practice for many years, the persistent challenge of resistance has cast a shadow on the overall efficacy of EGFR/VEGFR antibodies. The study underscores the complexities faced by drugs targeting various signaling pathways in clinical settings, acknowledging the multifaceted challenges encountered. It emphasizes the critical role of signaling transduction and the aberrant activation of pathways in CRC, shedding light on the intricate molecular landscape that contributes to the disease's progression. The study also highlights the current state of drug development, providing a snapshot of the drugs available at present. Looking forward, the study emphasizes the necessity for future research to focus on exploring efficient targets and drugs that can overcome resistance. The concept of rational combinations, informed by a deeper understanding of the intricate interplay of signaling pathways, is underscored as a key avenue for further investigation. Additionally, the study emphasizes the importance of developing rational models and considering optimal treatment schedules to enhance the effectiveness of therapeutic interventions.

In summary, this study not only reviews the historical and recent advancements in CRC therapeutics but also lays out a roadmap for future research directions. The call for a more nuanced and targeted approach, considering efficient targets, rational combinations, and optimized treatment schedules, reflects the ongoing commitment to advancing precision medicine in the challenging landscape of colorectal carcinoma treatment.

## Data Availability

Not applicable.
